# The first otologic surgery in a skull from El Pendón site (Reinoso, Northern Spain)

**DOI:** 10.1038/s41598-022-06223-6

**Published:** 2022-02-15

**Authors:** Sonia Díaz-Navarro, Cristina Tejedor-Rodríguez, Héctor Arcusa-Magallón, Juan Francisco Pastor-Vázquez, Jaime Santos-Pérez, Israel Sánchez-Lite, Juan Francisco Gibaja-Bao, Rebeca García-González, Manuel Rojo-Guerra

**Affiliations:** 1 Department of Prehistory and Archaeology, University of Valladolid, Valladolid, Spain; 2 Independent Archeologist, Zaragoza, Spain; 3 Department of Anatomy and Radiology, University of Valladolid, Valladolid, Spain; 4 Otolaryngology Service, Clinical University Hospital, University of Valladolid, Valladolid, Spain; 5 RadiodiagnoticsService, Clinical University Hospital, University of Valladolid, Valladolid, Spain; 6 Spanish School of History and Archaeology in Rome, Spanish National Research Council (CSIC), Rome, Italy; 7 Laboratory of Human Evolution (LEH), University of Burgos, Burgos, Spain

**Keywords:** Archaeology, Biological anthropology

## Abstract

Archaeological research in the Dolmen of El Pendón (Reinoso, Burgos, Spain) has brought to light the complex biography of a megalithic monument used throughout the 4th millennium cal. BC. The ossuary of this burial holds the bones of nearly a hundred individuals who suffered from diverse pathologies and injuries. This study presents the discovery of a skull with two bilateral perforations on both mastoid bones. These evidences point to a mastoidectomy, a surgical procedure possibly performed to relieve the pain this prehistoric individual may have suffered as a result of otitis media and mastoiditis. The hypothesis of surgical intervention is also supported by the presence of cut marks at the anterior edge of the trepanation made in the left ear. Furthermore, the results of this paper demonstrate the survival of the individual to both interventions. Given the chronology of this dolmen, this find would be the earliest surgical ear intervention in the history of mankind.

## Introduction

**Figure 1:**
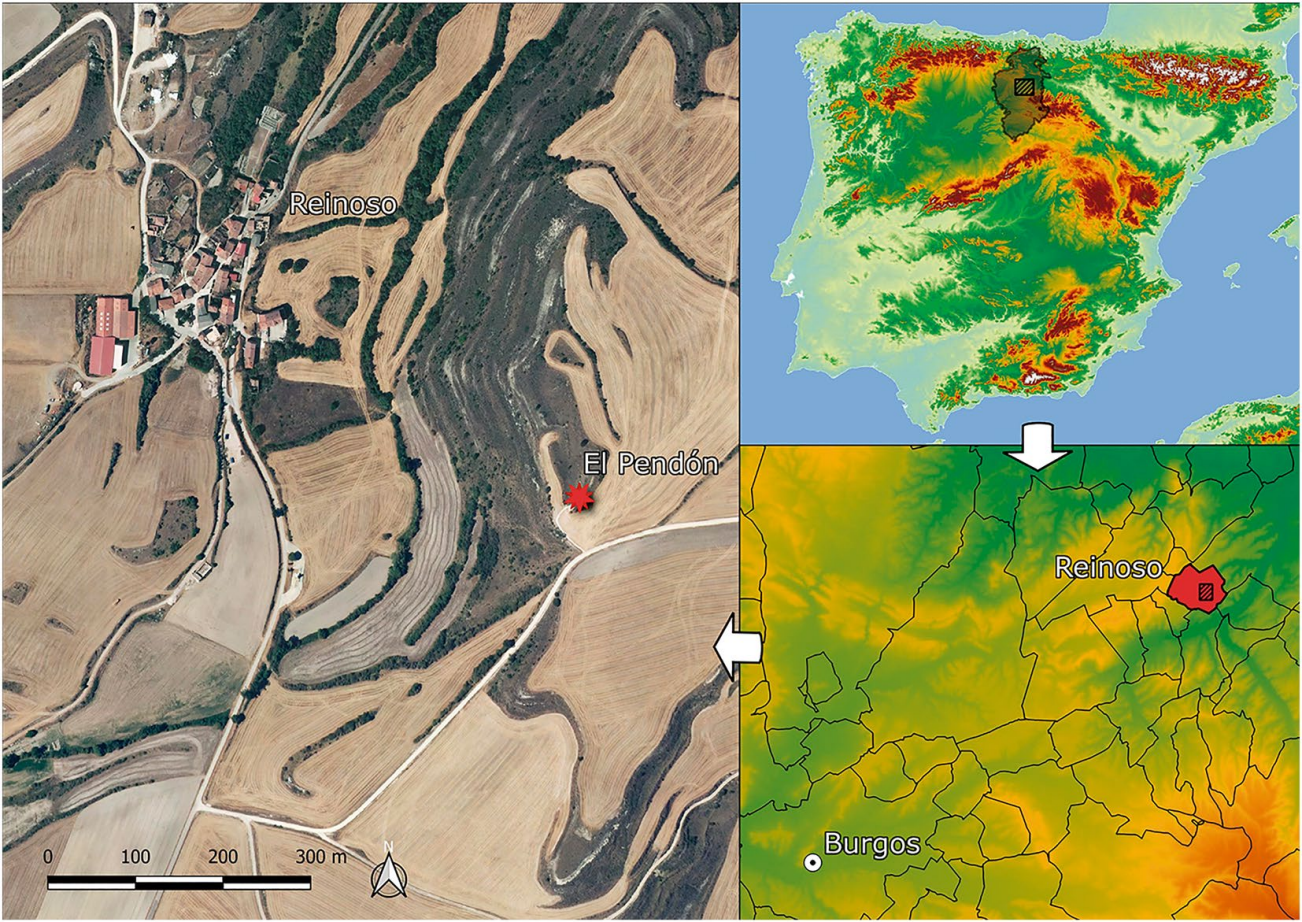
Map of the province of Burgos in the Iberian Peninsula and of the village of Reinoso and the Dolmen of El Pendón in its closest geographical context. The maps and the orthophoto were processed using the open software QGIS v.3.16 Hannover (available online https://www.qgis.org/es/site/).

**Figure 2:**
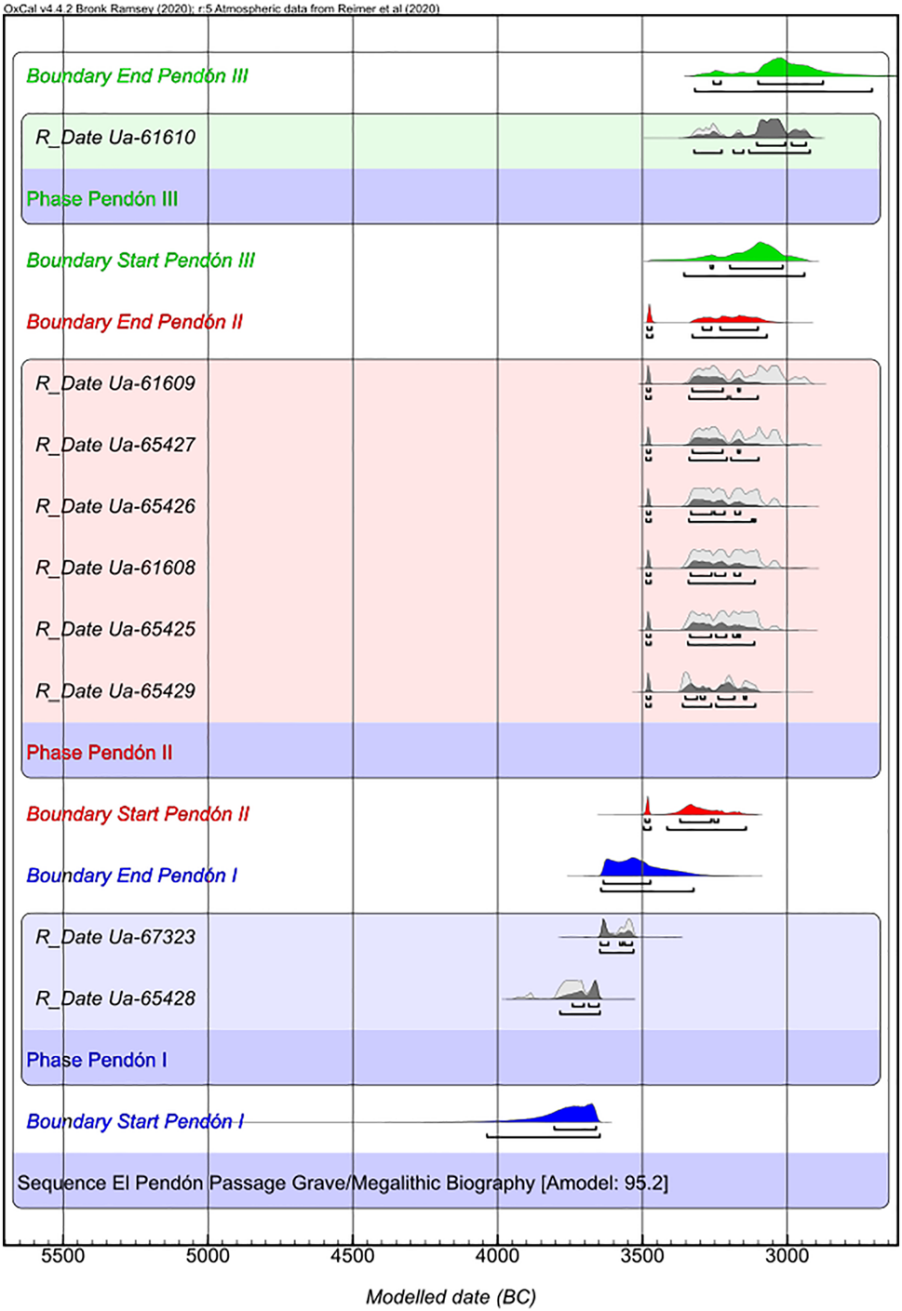
Bayesian model (*Phase sequential analysis*) of 14C dates from the Dolmen of El Pendón (ash grey curve = calibrated dates; dark grey = modelled dates). The estimated start and end boundaries are presented for each phase of megalithic activity. Please refer to Supplementary Text S2 for the agreement indices and other data about the chronometric analysis.

The archaeological excavation carried out since 2016 at the Dolmen of El Pendón (Fig. 1) have uncovered the complex biography of a megalith that, since its construction, went through several phases of use (Fig. 2, Supplementary Text S1 and Text S2) until its permanent abandonment as a tomb and its transformation into a commemorative monument—with completely different functions to the one it originally had (Supplementary Text S1, Text S2 and Video).

14C dates place its construction and first funerary uses to the beginning of the 4th millennium cal. BC (Fig. 2: Phase Pendón I, Supplementary Table S1). Its builders conceived a megalithic architecture widespread in the Iberian Peninsula that corresponds to a passage grave typology (Supplementary Fig. S2). It is composed of a funerary enclosure, a chamber formed by large upright orthostats, and an entrance passage of approximately eight metres of length. Around this internal structure, there is a mound made of stone and soil, which probably surpassed 25 m in diameter. However, the monument present look is completely different from the original because of an intentional and perfectly planned integral transformation process (Supplementary Text S1).

During the last quarter of the 4th millennium cal. BC, continuing with the usual behavioural pattern of megalithic tombs in the region^1^, the bodies were diachronically placed inside the megalithic chamber—almost one hundred in this second phase of use. This burial space was transformed into a collective ossuary where only a few anatomical connections were preserved over time, due to the development of ritual practices, such as the disarticulation and repositioning of the skeletal remains, which sought to break the individuality of the corpses buried there (Fig. 2: Phase Pendón II and Supplementary Text S1). In this phase, two different burial levels have been documented, characterised by the presence of repositioning practices as well as regrouping and selection of specific skeletal pieces (Supplementary Fig. S3 and S5). The exceptional find of the intentional assemblages of skulls and pelvises—documented in up to 15 different groupings—must be emphasised (Supplementary Text S1).

At the end of 4th millennium cal. BC (Fig. 2: Phase Pendón III and Supplementary Text S1), the monument was closured through a complex ritual that transformed its architecture. Only six of the big limestone orthostats that composed the original burial chamber are still standing. All the built structures of the passage have disappeared and the mound has been reduced to a pile of stones of a few metres in diameter. The site was no longer used as a tomb, even though it kept its symbolic value as a territorial reference, ceremonial centre, and a place of community congregation (Supplementary Text S1).

The ossuary features a collective and diachronic burial (Supplementary Fig. S1) in which human remains were repositioned in the same space where decomposition took place. Therefore, strictly speaking, it is a primary burial deposit, subsequently altered by an anthropogenic intentional action. This is demonstrated by the presence of some anatomical connections, such as the spine, the tarsal and carpal bones and the limbs, as well as the usual documentation of labile skeletal parts—small ear bones, hyoids or ossified thyroid cartilages, which would most certainly be lost during a hypothetical relocation of corpses (Supplementary Fig. S4).

This paper presents a comprehensive analysis of a skull from one of the burial levels in the second phase of this monument using an osteoarchaeological, chronostratigraphic, palaeopathological and histological approach. Its discovery has great scientific interest, since there are two perforations on both mastoid processes, most likely associated with a double mastoidectomy, with clear signs of survival (Supplementary Figs. S10 and S11).

## Results

### Description of the skull and its pathologies

**Figure 3:**
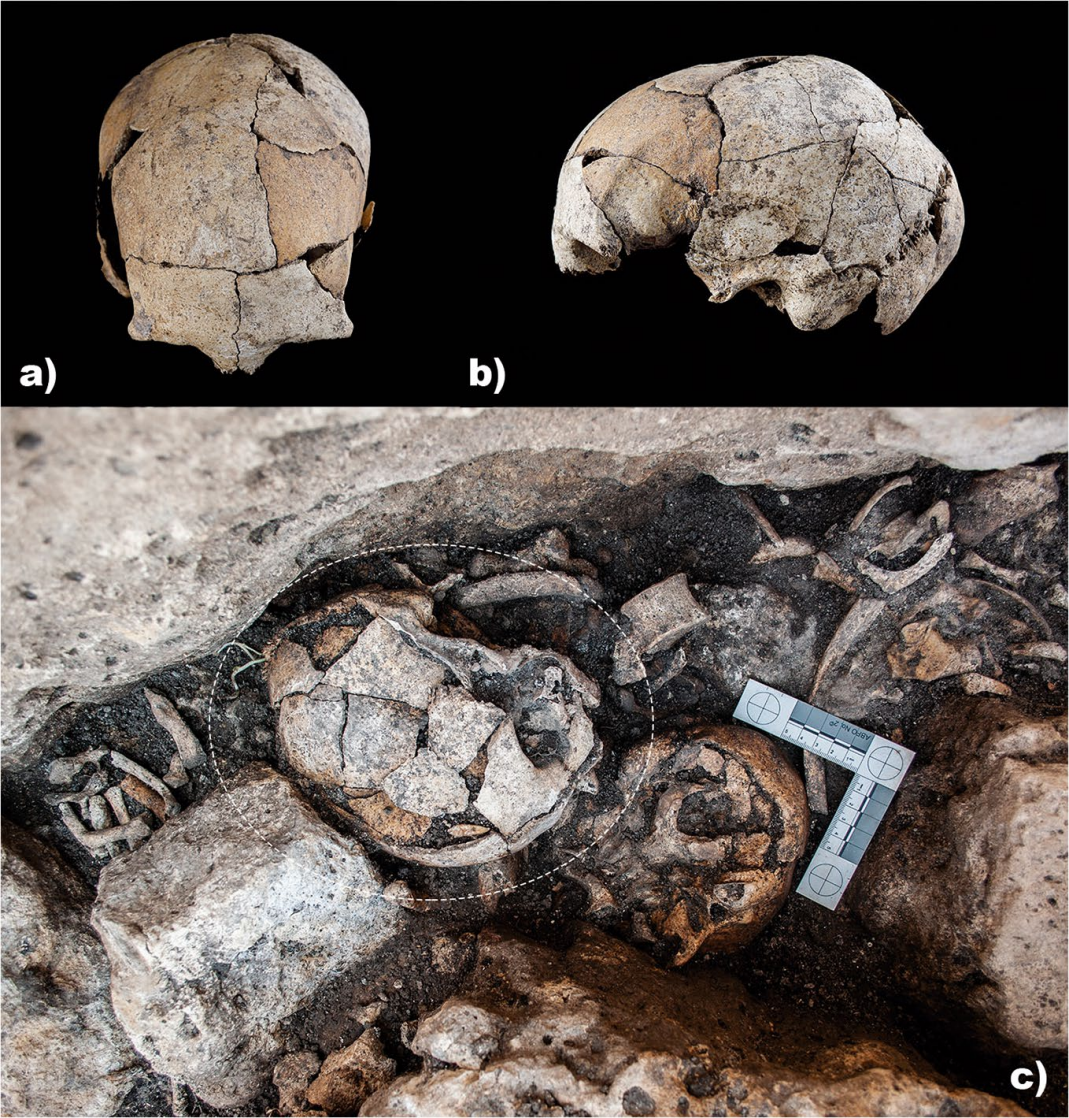
Skull under study found at El Pendón site. Superior: Frontal and lateral view of the skull (Photo: ÑFotógrafos Photography Study). Inferior: Skull with mastoidectomy in situ in the context of the megalithic ossuary.

This paper focuses on the finding of a skull from the Dolmen of El Pendón in July 2018. Its chronostratigraphic context corresponds to the second phase of use of this megalith. The skull was lying on its right side with the face pointing south, towards the entrance of the burial chamber (Fig. 3). It retained a complete neurocranium, including frontal, both parietal and temporal bones, and the occipital bone without the basilar section. Of the facial bones, the nasal bone, the zygomatics, and the lower region of the maxillary bone (without teeth nor alveolar cavities except for the first left molar) remained. Furthermore, root impressions were visible in the cortical surface of the frontal and parietal regions (Supplementary Fig. S6 and Video).

Analysis indicates that it is a mesocephalic skull that belonged to a woman, who probably died at an advanced age. While the obliteration of the cranial sutures is consistent with a middle-aged individual—35–50 years old, the loss of all the maxillary teeth long before death—given the alveolar reabsorption of the teeth and the loss of bone density—suggests an age range closer to elderly. This statement is based on the general good oral health of the community whose remains are deposited in the dolmen. Therefore, the loss of all teeth in life points to elderly individuals. The presence of elder individuals is confirmed by the documentation of fully ossified thyroid cartilages. This particular ossification is estimated to end at 65 years of age^2^.

**Figure 4:**
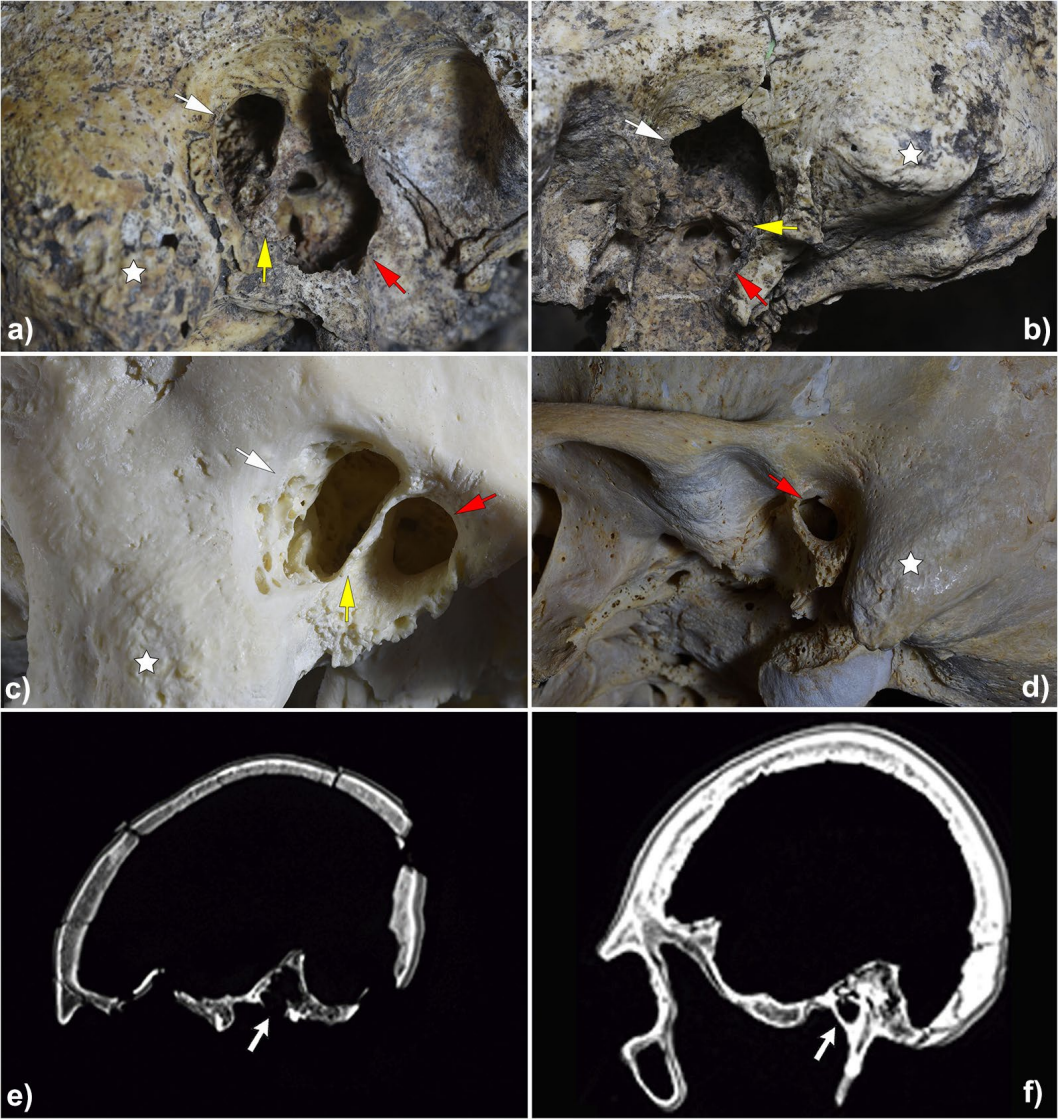
Computed tomography scans and details of both temporal bones of the skull under study and some samples of the comparative analysis. Superior: Details of external auditory region on the right (**a**) and the left (**b**) temporal bones of the skull under study. It is to be noted the deterioration of the tympanic cavity in both temporal bones due to taphonomic processes. Middle: Present-day skull with mastoidectomy performed by the students of the Faculty of Medicine of the University of Valladolid (**c**) and example of an archaeological skull without any pathology used for comparative analysis (**d**). Red arrows indicate the external auditory canal. White arrows point to bone erosion in the postero-superior part of the external auditory canal due to the mastoidectomy. Yellow arrows indicate the *scutum*—thin bony spur that is formed by the superior wall of the external auditory canal and the lateral wall of the tympanic cavity. *Mastoid process. Inferior: Computed tomography (CT) scans of para-sagittal sections at right middle ear level—the arrows point to the middle ear—from the skull under study (**e**) and from a present-day skull without pathology (**f**).

The external auditory canal is enlarged on both sides in a postero-superior and inferior direction, connecting the mastoid cells and the tympanic cavity with the outside (Fig. 4). The edges are smooth and round; on the right side, its diameter is 12 mm, while on the left side it is 9 mm. No fracture zones, fissures, or bone calluses are visible on either side. The inner surface of both cavities shows typical speculated bone formations, which reflect common bone reabsorption changes in inflammatory mastoid processes^3,4^. However, both cavities reflect no important lack of mastoid pneumatisation, something common in individuals suffering from middle ear inflammatory pathologies in childhood^5^, suggesting a late onset of an underlying disease. It is important to emphasise that the bony wall separating the ear canal from the mastoid—*scutum*—has been preserved on both cavities (Fig. 4).

Signs of bone regeneration and remodelling are evident. Traces of mastoiditis or mastoid abscesses found in palaeopathological analyses of ancient skulls show major osteolytic defects without repair signs, which, in the absence of medication or adequate surgical drainage, often have a tragic end^6^. Nevertheless, the surface histological analysis show that signs of bone remodelling are manifest in the performed trepanation of this skull, thus evincing the survival of the individual. Remodelling field are binary features presenting forming or resorbing surfaces^7^. During adulthood this process is a “secondary remodelling”, in which bone resorption and bone deposition occur at the same site replacing old and damaged bone in highly regulated cycle^8^. In the dry bones we can detect four distinctive surfaces: resorptive, characterized by osteoclasts and resorption lacunae; depository, characterized by osteoblasts; resting—or neutral, characterized by cells performing no bone activity and remodelling reversals, which are interfaces between fields of resorption and deposition^9^.

Our results shows that bone resorption and resting are the only activity states present over the area of intervention in the left ear (Supplementary Fig. S10). The resorptive surface is identified for the presence of Howship’s lacunae and could be related to the replacement of damaged bone due to infection. Indeed, we do not find any signal of pathological bone. The most plausible interpretation of these results is that after the intervention this woman survived, since the resorption is ongoing, but the pathological bone has been eliminated. In contrast, in the right ear clearly visible resorption areas as well as a bone deposition area provides evidence that remodelling reversal is ongoing (Supplementary Fig. S11). The resorption phase is approximately two weeks in duration. After this phase, the reversal one lasts approximately 4 to 5 weeks^10^. Thus, the presence of a well-defined reversal line in this right ear means that this woman survived to the intervention.

### Differential diagnosis

The hypothesis proposed in this research is that the individual to whom the skull belonged was probably surgically intervened on both ears, with an undetermined period between both interventions. Based on the differences in bone remodelling between the two temporals, it appears that the procedure was first conducted on the right ear, due to an ear pathology sufficiently alarming to require an intervention, which this prehistoric woman survived. Subsequently, the left ear would have been intervened; however, it is not possible to determine whether both interventions were performed back-to-back or several months, or even years had passed. It is thus the earliest documented evidence of a surgery on both temporal bones, and, therefore, most likely, the first known radical mastoidectomy in the history of humankind.

A well documented disease in palaeopathological studies of ancient skulls are cholesteatomas^11^. A cholesteatoma is a destructive injury of the temporal bone, which tends to expand and progressively erode the adjacent structures, causing hearing loss, vertigo and intracranial complications; it is treated surgically^12^. A rare type of cholesteatoma is the congenital cholesteatoma, characterised by the presence of epithelial embryological remains in the middle ear, generally associated with well-pneumatised mastoids in young patients, where the destruction of the tympanic cavity predominates^5^. Acquired cholesteatomas are more frequent; they are associated with sclerotic or diploic mastoids, in which epithelial remains are introduced in the middle ear through tympanic perforation or invagination. This tends to occur in postero-superior quadrants, starting with the initial erosion of the *scutum* or the bony wall of the atticus^13^. It is also known as the external auditory canal cholesteatoma, which is usually unilateral and characterised by an epithelial accumulation that may evolve into extensive temporal bone erosion in patients with a history of injuries or surgery. Its spontaneous appearance is quite rare^14^. Lastly, malignant external otitis, histiocytosis, or tumours can produce extensive bone destruction. However, they are rarely bilateral and often cause the premature death of the individual.

Here, acquired cholesteatoma of the middle ear must be ruled out, since the *scutum* is present on both temporal bones. Other diseases, such as malignant external otitis or temporal bone tumours are also discarded a priori, since they are rarely bilateral and generally result in an untimely death, for which the documented bone remodelling previously described on both temporals would be impossible. Finally, a bilateral congenital cholesteatoma or one from the external auditory canal—both rare—can hardly be the cause of the mastoid condition found in the tympanic cavity that led to the performance of this pioneering surgery.

### Surgical instruments

**Figure 5:**
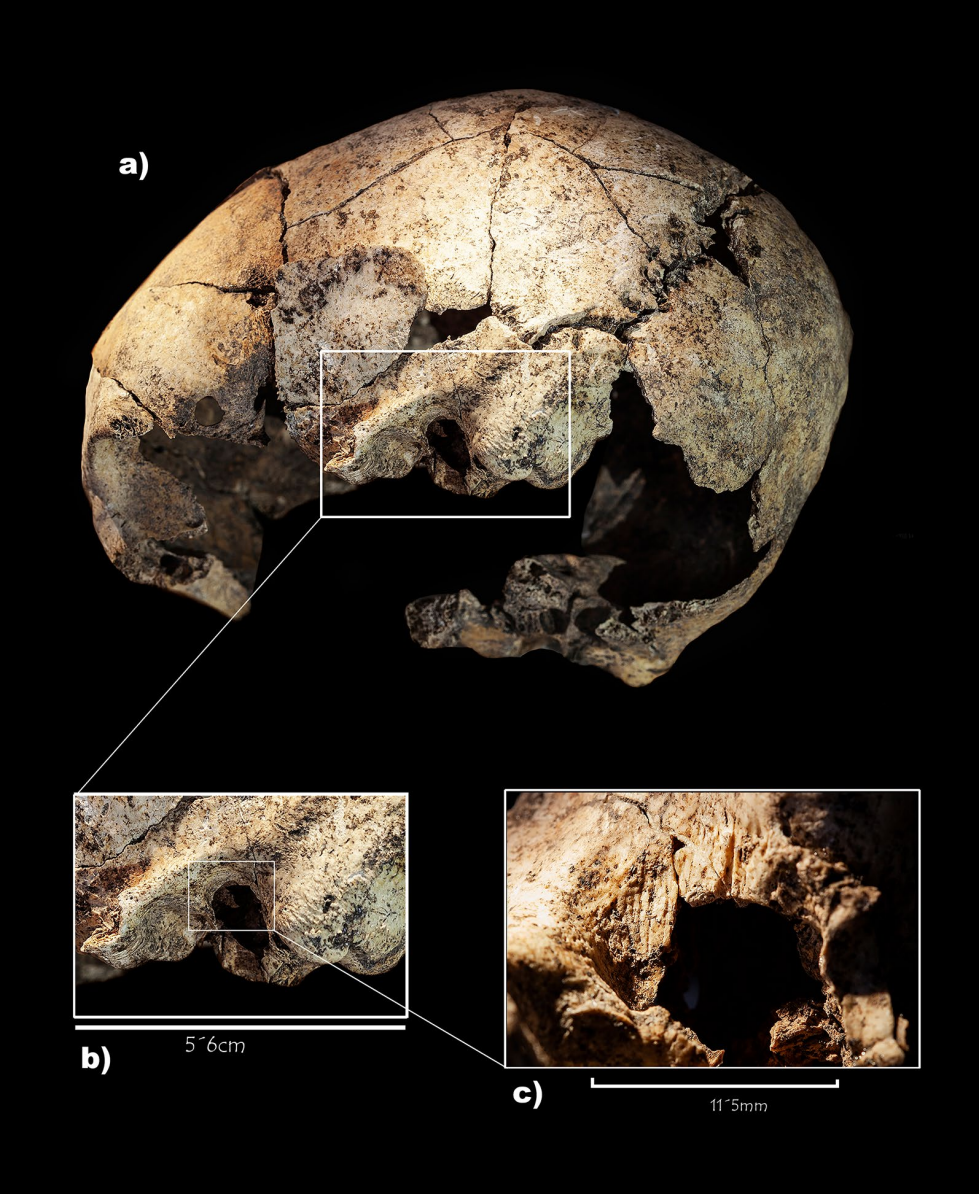
Set of cut marks identified on the left temporal bone of the skull under study. Lateral view of the left side of the skull (**a**), detail of the left temporal bone with the otological surgery (**b**), and enlarged image of the cut marks located at the anterior edge of the surgical trepanation made in the left ear, next to the mastoid process (**c**).

Together with the above-mentioned macroscopically visible evidences in the temporal bones, seven cut marks at the anterior edge of the surgical trepanation made in the left ear have been identified. They are parallel, short (2–4 mm) and linear with a clear triangular or "V" section. However, these marks are not visible on the right side, probably due to the bone remodelling process that was ongoing (Fig. 5).

**Figure 6:**
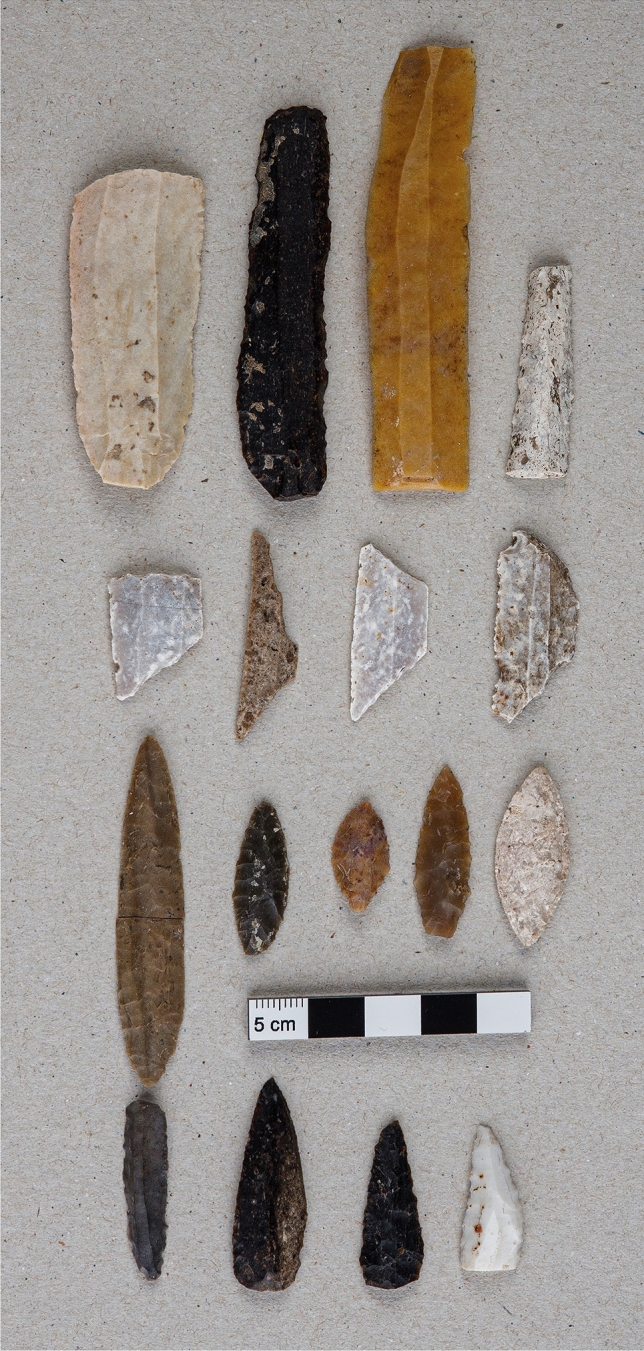
Selection of a set of flint lithic tools—blades, geometric microliths, and arrowheads—from the El Pendón ossuary. In the line below, four lithic tools that were submitted to a 'blind' analysis by a specialist in traceology and use-wear analysis (Supplementary Text S3).

This finding is further strong evidence that this is the earliest mastoidectomy documented to date. Given the pre-metallurgical chronology of the site, this surgical intervention had to be performed with a lithic instrument. Several pieces were deposited as grave goods or ritual offerings next to the dead. The most important were tools made of flint of different provenance, of which several typologies have been identified: simple and retouched blades of different sizes, geometric microliths and arrowheads of different shapes (Fig. 6).

A blind traceological analysis was conducted on a set of lithic tools from the dolmen with the purpose of identifying the possible technique or tool that allowed performing this particular surgery. One piece showed signs of having been used for butchering and probably came into contact with bone material: a flint blade, 31 mm in length and 7 mm wide at its distal end, with simple direct retouches that formed slight indentations (Fig. 6). The traceological analysis has revealed that after being extracted from the core the blade was subjected to heating that did not exceed 300/350 °C, given the lack of fire-cracks and other type of heat treatment marks (Supplementary Fig. S9 and Text S3).

## Discussion

The findings suggest that the intervention could be caused by an acute otitis media, further complicated with mastoiditis, and caused a sub-periosteal abscess with rupture of the mastoid cortex. The ensuing infection affects the mucous membrane of the tympanic cavity and can spread to the pneumatised spaces of the mastoid process. Its hive-like structure provides an ideal environment for the proliferation of pyogenic bacteria^15^. These infections are often caused by the proliferation of bacteria or other pathogens in the middle ear. Mastoiditis per se is a consequence of otitis media and it is clinically diagnosed when the infection spreads from the mucous membranes to the underlying bone^3^. If left untreated, the infections could result in hearing loss and even meningitis. In a prehistoric context, this pathology would be detectable since it presents itself as a fluctuating and painful retroauricular mass. The first otological surgeon executed a systematic cleaning and drainage process by removing the affected bone and connecting the mastoid bone with the tympanic cavity. This procedure was facilitated by the prior existence of a Gellé fistula—destruction of the posterior wall of the external auditory canal. All these facts paved the way for the pioneering mastoidectomy known in the history of humankind.

Middle ear infections are common modern human diseases. They were already known in ancient Egypt and Mesopotamia, as evidenced by some papyri^16^. The palaeopathological analyses had already identified these illnesses in the Palaeolithic period^17,18^, although there is greater evidence from the Neolithic period onwards^4,19–23^.

Mastoidectomy was a relatively common surgical procedure for the treatment of acute ear infections in the pre-antibiotic era. The first descriptions of mastoid surgery, dated to the seventeenth century, were implemented by Johannes Riolanus the Younger^24^. The earliest documented osteological evidence of mastoidectomy comes from the island of Thassos during the Proto-Byzantine period^25^. In addition, an eleventh century skull with mastoidectomy was found in Croatia^26^. Another two possible cases were identified in an Italian ossuary from the late eighteenth century and early nineteenth century^27^. Finally, a 19th or twentieth century skull recovered from the Assistens Kirkegård Cemetery (Copenhagen) also shows evidence of an ear surgery^28^.

Surgery in other areas of the skull is one of the earliest surgical procedures. Cranial trepanations have been documented with Mesolithic chronologies in Ukraine, Portugal, and Northern Africa^29,30^, even though most prehistoric cases date back to the Late Neolithic and Bronze Age. This intervention is well documented in Europe, and the Iberian Peninsula stands out with 184 identified cases of trepanation in 135 skulls^31^. The case of the Dolmen of Las Arnillas is exceptional (Sedano, Burgos). Located just 40 kms in a straight line from the Dolmen of El Pendón, up to six trepanations were carried out in five skulls, all of them with clear signs of survival^32^. Although there are no documented lesions or pathological evidences that could justify such surgical procedures, an increasing number of researchers informs of the presence of otological pathologies in individuals with trepanations, generally mastoiditis^33–38^.

This is a plausible interpretative hypothesis to confirm that the evidence presented in this paper corresponds to the earliest documented otological surgery in History. In this case, the prehistoric surgeon located the focus of the problem—probably because the infection was evident to the naked eye—and successfully intervened, as proven by the bone regeneration observed in both mastoid bones (Fig. 4 and Supplementary Figs. 10 and 11).

The intervention itself would have consisted of a progressive circular and abrasive drilling causing unbearable pain under normal conditions. Thus, in order to carry out this surgery, the affected individual had to be either strongly restrained by other community members or previously administered some psychotropic substance with the purpose of relieving the pain or losing consciousness. There are references to the use of plants with natural analgesic and antibiotic properties in prehistoric times^39^, as well as psychoactive drugs like opium^24^ or hyoscyamine^40^, as it is the case in two skeletons from Can Tintorer^41^ (Gavà, Barcelona).

Despite the above-mentioned evidences of cut marks (Fig. 5), it is difficult to conclude the type of tool used to remove the bone tissue, most likely a sharp instrument with a circular movement. The researchers cannot decisively state that the thermally altered flint blade presented above, whose use-wear traces are compatible with the ones used on bones, was the tool employed in this intervention (Supplementary Fig. S9). However, the results of the traceological analysis suggest that it could have served as a cautery, thus becoming a surgical tool used in a healing procedure for organic tissues through the application of high heat (Supplementary Text S3). Several studies demonstrate the use of stone instruments heated with fire as tools to cauterise wounds and to perform trepanations, in primitive villages of the Canary Islands^42^ and among the megalithic populations of the region between the Oise and Sena rivers to the north of Paris^43^. L. Manouvrier, in a bold and even romantic way, based on the many trepanations documented in megaliths in this French region, surmised the existence of remarkable chirurgiens who travelled around offering their wisdom and skill^43^.

## Methods

### CT and virtual reconstruction of the skull

This study analysed and photographed the skull with a Zeiss surgical binocular magnifying glass (Model 800). In addition, a multi-slice computed tomography (CT) of the entire skull was conducted, comparing it with the skull of a present-day individual with a healthy ear (Fig. 4), using a Toshiba tomography with 64 detectors (Aquilion Model); 2 mm slices were made every 1.5 mm. The virtual 3D reconstruction was generated using *3DSlicer*© software. Further, the 3D photogrammetric model was developed from 267 photographs taken with a Nikon D750 reflex camera and were later processed using the *Agisoft Metashape Professional* software (Supplementary Fig. S6). The images of the skull included in this paper were taken with that reflex camera and a TAMRON 90MMSP F2.8 macro lens.

### Osteology

The sex estimation was based on the analysis of morphological and morphometric features of the skull, following the criteria set by Buikstra and Ubelaker^44^. To determine the age at death, we used the analysis of the degree of ectocranial obliteration, according to the method employed by Meindl and Lovejoy^45^, given the absence of the teeth and postcranial skeleton of the individual (Supplementary Text S1).

### Histological analysis

The surface histological analysis were performed in the Laboratory of Human Evolution (LHE) of the University of Burgos. Negative impressions of surfaces of both temporal bones were made with silicone cast material (President, light body micro System). These silicone molds were used to generate resin casts by placing polyurethane resin (Feropur Pr-55, Feroca) onto negative impression and letting it dry. These replicas were then coated with gold. The gold-coated high-resolution replicas were imaged using a scanning microscope JEOL JSM-6460lv in vacuum mode at 100×.

### Chronometric analyses

The chronometric analyses and the calibration and modelling graphs of the 14C dates presented were done with OxCal v4.4.2 software (available online http://c14.arch.ox.ac.uk/)^46^, applying the IntCal20 atmospheric calibration curve^47^ (Supplementary Text S2).

### Traceological analysis

The use-wear and traceological analysis of the lithic tools were conducted as a ‘blind test’ to avoid conditioning the results based on the characteristics of the archaeological site. The specialist researcher was completely unaware of the provenance of the sample under study. Two blades and two arrowheads, both made of flint, were selected for this analysis (Fig. 5 and Supplementary Text S3). Its study involved the combined use of a Leica MZ16A binocular magnifying glass, with 10× to 90× magnification, and an Olympus BH2 metallographic microscope, with objectives ranging from 50× to 400× magnification. The images were taken with a Canon 450D camera and processed with a photographic software called Helicon Focus v.4.62.

## Supplementary Information

Supplementary Information.

Supplementary Video 1.
