# Supplementary material for: The first otologic surgery in a skull from El Pendón site (Reinoso, Northern Spain)
Source: Sci Rep. 2022 Feb 15;12:2537. doi: 10.1038/s41598-022-06223-6 (PMC8847418; doi:10.1038/s41598-022-06223-6)
Supplement: Supplementary file 1 — Supplementary Information. [file 41598_2022_6223_MOESM1_ESM.pdf]

SCIENTIFIC REPORTS

Supplementary Information for

The first otologic surgery in a skull from El Pendón Site (Reinoso, Northern Spain)

Sonia Díaz-Navarro, Cristina Tejedor-Rodríguez, Héctor Arcusa-Magallón, Juan Francisco Pastor-Vázquez, Jaime Santos-Pérez, Israel Sánchez-Lite, Juan Francisco Gibaja-Bao, Rebeca García-González Manuel Rojo-Guerra

*** Corresponding author. Email: marojo@uva.es**

This PDF file includes:

Supplementary Texts S1-S3

References for Supplementary Texts (49-60)

Table S1 – S3

Fig. S1 – S11

Supplementary Video

Supplementary Video Legend

Supplementary Texts

Text S1. Chronostratigraphy and osteoarchaeology of El Pendón

Text S2. Bayesian chronometric analysis on 14C dates from El Pendón

-‘Sequential phases’ Model

-‘Contiguous phases’ Model

Text S3. Use-wear analysis report on a selection of lithic elements from El Pendón

Text S1. Chronostratigraphy and osteoarchaeology of El Pendón

The archaeological work at the Dolmen of El Pendón is still in progress. The results so far, have revealed the complex biography of this megalithic monument, which is characterised by long-lasting processes of rite-funerary use (Fig. 2); unique post-mortem practices of selection and reorganisation of human remains; and an exceptional enclosure and transformation event of the monument. Preliminary osteoarchaeological analysis (pending completion of the excavation of the lower levels of the chamber and the passage), confirms that almost one hundred individuals of both sex and all age groups were buried in this dolmen (Supplementary Fig. S1). The estimate of the minimum number of individuals (MNI) is 90 after counting the mandibular fragments and classifying them according to age. Sex has been estimated for 40 individuals (16 females; 10 males; 7 probably females; and 7 probably males) based on the morphological and morphometric analysis of the pelvic bones[49]. Dental eruption[50] and wear analysis[51] in the mandibles were used to estimate the age. The findings indicate one neonatal; six individuals from the Infant I category (1-6 years old); 10 from Infant II (7-12 years old); 14 subadults (13-20 years old); 29 young adults (21-35 years old); 20 mature adults (36-50 years old); 8 undetermined adults, and two elder individuals (>50 years old).

The first phase of use refers to chronologies from the first half of the 4th millennium cal. BC, both for the original architectonic typology of a passage grave[52] and for the 14C dates obtained from human bones found along the passage (Supplementary Table S1), especially in the entrance area or atrium (i.e. the area furthest from the chamber) (Supplementary Fig. S2). The location and appearance of these bone remains reflect the action of severe post-depositional processes such as root marks and exposure to the weather. It alludes to the hypothesis that they were part of a previous ossuary housed in the burial chamber itself, which was ‘emptied’ and its remains manipulated and repositioned in other areas of the megalith to obtain space for a new burial repository.

The 14C dates from this new ossuary repository place the second phase of use of the dolmen, in the last quarter of the 4th millennium cal. BC (Supplementary Table S1). This monument was again used intensively as a collective and diachronic burial during this phase, which only lasted two centuries. The large density of skeletal remains recorded in the ossuary is proof thereof, which spreads in a homogeneous way throughout the megalithic chamber. Even though there seems to be no difference a priori, thanks to the systematic and scrupulous methodology of excavation and recording, two burial levels have been identified, that were stratigraphically different although not chronologically.

The lower level of this ossuary is recorded as stratigraphic unit 4 (SU 4), and despite still being under excavation, it is the one with the highest density of buried human skeletal remains (Supplementary Fig. S3). Practically all anatomical regions are represented with a rather homogeneous percentage distribution (Supplementary Fig. S4). The most common bones are the ones from the thorax, spine, hands, and feet, mainly because of their greater frequency and high fragmentation. The presence of some partial anatomical connections and of intentional assemblages of certain skeletal pieces, such as mandibles, sacrum, or long bones, among others, stand out in this level, as well as the large concentration of children remains, especially considering their usual under-representation in these types of contexts[53,54]. In contrast to the virtual absence of complete skulls in this level, more than 75% of long bones were recorded in it (Supplementary Fig. S4). However, it should be noted that the long bones count is quite inferior to the one expected according to the estimated MNI, a fact that could be the result of recurring and intensive manipulation and repositioning practices of human bones that occurred throughout this megalith biography.

The lower ossuary is covered by a level of stones (SU 2), formed by large limestone and soft sandstone slabs. On that, another level of human bones was deposited buried, which could be interpreted as the recreation of an ossuary or a fake ossuary (SU 18). Firstly, bone density is not as high as in the previous case and the percentage distribution of the anatomical areas is neither as diverse nor homogeneous (Supplementary Fig. S4). There is also an accumulation of remains towards the periphery of the megalithic chamber, as opposed to the even distribution of the above-mentioned burial level (Supplementary Fig. S5). Once again, the largest group of bones are the ones from the thorax, spine, hands and feet. The large representation of the cranial skeleton and pelvic girdle are noteworthy as they form 88% of the skulls and 64% of the complete or semi-complete pelvic bones recovered from the burial chamber. These unique features are caused by the fact that this burial level is the result of a succession of events of selection, manipulation, and reorganisation of human bones from the lower ossuary (SU 4), before being covered by a layer of stones (SU 2). Such events did not occur randomly or accidentally but were intentional and perfectly planned. The diversity of ritual behaviour patterns that have been identified when carrying out this type of practice is proof of this. This is the case of multiple assemblages and superimpositions of the same type of skeletal pieces, such as mandibles or the well-known ‘skull nests’[1]. One of the reorganisation practices of bone remains, exclusive to the Dolmen of El Pendón to date, is the intentional arrangement of a skull on one or two coxal bones, disposed as a bed. This type of association has been documented up to 15 times, mainly in the periphery of the burial chamber (Supplementary Fig. S5). It was in this ossuary level that the skull of this study

was found (Supplementary Fig. S6), which was superimposed on another adult skull with peri mortem blunt force trauma and laid out at the same time over a right pelvic bone, forming the grouping identified as skull-pelvic assemblage Nº 12 (Fig. 3). This upper ossuary level (SU 18) was also sealed by a level of large limestone stones (SU 19).

The third phase of use took place during the transition from the 4th to the 3rd millennium cal. BC (Supplementary Table S1). It is actually a very complex closure and transformation process of the monument, involving the total dismantling of the passage structure, part of whose orthostats were placed inside the chamber (SU 7). This fact allowed the preservation of a large part of the ossuary in situ, without barely any modern alterations. Even though exceptional examples of closure of other megalithic monuments have been documented in nearby geographical areas, involving the celebration of complex ritual practices[55] and/or the total transformation of the megalithic architecture and functionality[56], the process documented at El Pendón is unique. After its closure, this dolmen ceased to fulfil its original role as a collective tomb, maintaining and even increasing its already important symbolic value as a territorial reference, ceremonial centre, and place of community gathering for generations.

Text S2. Bayesian chronometric analysis on 14C dates from El Pendón

This case study is based on nine 14C dates from the Dolmen of El Pendón (Supplementary Table S1). Although it is a small sample, its chronometric analysis is perfectly feasible, since representativeness was considered for its selection, more or less balanced, from all the possible phases of use that were documented in this megalithic monument.

All samples correspond to short-lived events, specifically human and animal remains, whose collagen levels were all analysed. In no case is the standard deviation greater than 45, which allows a better adjustment of the calibrations (Supplementary Table S1). The information about the archaeological context of each sample, has allowed the implementation of different chronometric analyses, in order to chronologically characterise the biography of this dolmen. In fact, the individualised study of each one of the 14C date and its associated archaeological information has been fundamental to the analyses, since megaliths are sites that have been recurrently used, so the provenance of any material evidence may be altered by subsequent occupation events.

After this first contextual analysis, each of the 14C date was individually calibrated, to obtain a preliminary view of the chronological behaviour of the activities that took place in this dolmen (Supplementary Table S1).

In order to try to accurately define the phases of use and estimate its chronological boundaries with a high statistical probability, other proper analytical methods of Bayesian statistics have been implemented. In this type of chronometric analysis, it is possible to include aprioristic information about archaeography, such as the relationship of the sample with the use sequence of the site or its association to specific artifacts and/or ritual-funerary practices. To perform these analyses, we worked with modelled dates, which decreased the degree of statistical uncertainty resulting from the margins of error of usual calibrations. The models presented have acceptance rates well above 60%, either regarding the agreement of the general model or each one of the analysed dates (Amodel/Aoverall), providing valid results for interpretation (Supplementary Tables S2 and S3).

Two different types of Phase analysis have been applied based on these assumptions, using a multi-phase model (in this case are three phases), with no pre-established temporal order[57]. The difference between the two types of analyses lies in the relationships and discontinuities between phases. In both cases, Span and Interval commands have been used to calculate the period between the estimated start and end boundaries[47], that is, the duration of each phase of use. These commands use different parameters to calculate the interval probability distribution of a phase, sequence, or group of events. While the Span command measures the difference between the earliest and the most recent event in a group of 14C dates, the Interval command makes the particular calculation including events that were not directly dated. Therefore, both tools are compatible and their results complementary (Supplementary Tables S2 and S3).

It should be noted that other chronometric analyses have been tested, starting from a model with only two phases, unifying the 14C dates associated with the second and third phase of use. Even if the resulting models showed an acceptable agreement index, they were much lower than those from the other models. Therefore, since it was considered the weakest hypothesis, both in chronometric and archaeological terms, it was decided not to include its results in this paper.

- ‘Sequential phases’ Model

It is a multi-phase chronometric analysis, in which the phases are considered independent from each other, with the possibility of a chronological gap between them. The objective of this model is to estimate the start and end of each phase. The graph (Supplementary Fig. S7), in addition to each one of the modelled 14C date, shows the chronological intervals in which the estimated start and end boundaries for each phase of use.

The results of this analysis (Supplementary Table S2) mark the estimated start of the biography of this dolmen at the beginning of the 4th millennium cal. BC (4037-3648 cal. BC 2σ), with a first phase of use that lasted several centuries. After quite a clear and well-defined no-activity period

(between 59-294 yr. 1σ), the second phase of use took place, which was much shorter (between 0-84 yr. 1σ), a fact that was already intuited a priori given the homogeneity of the 14C dates included in this phase (Supplementary Fig. S7). Lastly, the so-called third phase of use is a complex sequence of archaeological events that resulted in the complete transformation of the megalith. The sample associated to this phase comes from a small pit and dates to the last events that took place during the dolmen closure and remodelling process during the transition from the 4th to the 3rd millennium cal. BC (3322-2922 cal. BC 2σ).

- ‘Contiguous phases’ Model

This second model assumes the proposed phases as consecutive and estimates the transition period between them. In this case, a probability distribution of the estimated chronology for the transition interval is also presented (Supplementary Fig. S8).

The results obtained from this analysis are very similar to those of the previous analysis, except for some small differences (Supplementary Table S3). The novelty is the estimate of the start limit of the first phase of use, 4469-3652 cal. BC 2σ, slightly extending both the duration of each phase or the intervals that separate them.

The results obtained with both models appear to reinforce the hypothesis of the development of megalithic activity in this dolmen by different phases of use and, at the same time, both complement each other. In both cases, it indicates a longer first phase of use that could span over several centuries. After a period of at least two hundred years of no-activity, burial practice was resumed in this dolmen in a very intense way, though briefly (lasting one or two centuries). Taking into account the overlap between the estimated end of the second phase of use and the start of the third one, the closure process that completely transformed this monument and put an end to its use as a collective tomb could have taken place shortly after the last burial uses.

Text S3. Use-wear analysis report on a selection of lithic elements from El Pendón

The following is the full report of the results of the use-wear and traceological analyses of the four lithic artifacts that were selected (Fig. 6), performed by the research specialist and co-author of this paper, Juan Francisco Gibaja-Bao.

1. Flint blade retouched on both sides (reference code: 19.03.18.6336) Its dimensions are 31x8x3 mm. The presence of an intense gloss on the inside the retouch (Supplementary Fig. S9) indicates that it was caused after heating the blade. This fact caused a matt look of the external surface, while on the inside there was still an intense gloss. The absence of another type of heat treatment

marks, such as fire-cracks, splintering of surface, or patina, suggest the heat treatment did not surpass 300º-350ºC[58-60]. It is not possible to ensure that the heating was intentional. However, if it was, it should have been carefully conducted, since more heat or a sudden temperature change would fracture it. The existence of meat polish was identified in some areas of both edges, with areas with compact polish and striation marks due to contact with the bone. Probably, this group of use-wear traces is the result of an activity of butchering. These traces have been documented in the more external areas of the edge, quite precisely, and especially on the right edge's distal corner of the studied flint blade. Usually, these pieces of evidence are difficult to identify, but the compact polish caused by the contact with the bone allows this determination. Such a statement relies on experimental archaeology programs that offer comparative references to address the analysis of the archaeological remains.

2. Distal fragment of flint blade with retouch on both sides (ref. 18.20.0.3164). Its dimensions are 25x11x4 mm. Its analysis has been impossible because of the intense gloss presented on the surface, which could also be the result of the heat treatment of the core that produced the blade.

3. Flint arrowhead, with an elongated rhomboidal morphology, elaborated from a blade blank (ref. 16.20.0.2160). Its dimensions are 37x13x3 mm. It has neither impact fractures nor striation marks because of the impact of a projectile. On the other hand, it presents rounded areas on different parts of the middle-proximal sides, which are related to contact with an abrasive matter such as hide. Possibly, such marks resulted from the bindings used for tying the arrowhead to the shaft or because of the contact with the interior of a quiver.

4. Flint arrowhead, with a triangular morphology, elaborated on a blade (ref. 17.10.18.1801). While there is a feather type impact scar in the apical area, the proximal area also shows a bending fracture. This evidence suggests that the artifact was used as a projectile after some of the alterations, as it did not lose its efficacy. As in the previous case, it presents some intense rounded areas in both lateral sections that could have been caused by the bindings of the tool or by keeping it into a quiver.

References

49. Ferembach, D., Schwidetzky, I. & Stoukal, M. Recommendations for age and sex diagnosis of skeletons. *J. Hum.Evol.* **9**, 517-549 (1980). https://doi.org/10.1016/j.jchb.2005.07.002

50. Ubelaker, D. H. *Human Skeletal Remains: Excavation, Analysis and Interpretation* (Smithsonian Institute Press, Washington DC, 1978).

51. Zoubov, A. A. *Odontología. Métodica de las investigaciones antropológicas*. (Nauka, Moscú, 1968).

52. Tejedor-Rodríguez,C. & Rojo-Guerra,M. A. "An approach to the Megalithic Architectures in the Douro Basin: some chrono-typological remarks and examples about the use of different lithologies" in *Megaliths and Geology*, R. Boaventura, R. Mataloto, A. Pereira, Eds. (Archaeopress, Oxford, 2020), pp. 109-134.

53. Fernández-Crespo, T. & de la Rúa, C. Demographic evidence of selective burial in megalithic graves of northern Spain. *J. Archaeol. Sci.* **53**, 604-617 (2015). https://doi.org/10.1016/j.jas.2014.11.015

54. Díaz-Navarro, S. Aproximación a la composición demográfica de los sepulcros megalíticos de la Submeseta Norte española. Un enfoque desde la osteoarqueología. *Espacio, Tiempo y Forma*. Serie I, Prehistoria y Arqueología. 14, 1–32 (2021). https://doi.org/10.5944/etfi.14.2021.29149

55. Tejedor-Rodríguez, C., Rojo-Guerra, M. A., Garrido-Pena, R., García-Martínez de Lagrán, I. & Palomino-Lázaro, A. L. Biografía' de un monumento megalítico: fases de uso y clausura en el dolmen de El Teriñuelo (Aldeavieja de Tormes, Salamanca). *Zephyrus*. **79**, 39-61 (2017). https://doi.org/10.14201/zephyrus2017793961

56. Rojo-Guerra, M. A., Garrido-Pena, R. & García-Martínez de Lagrán, I. Tombs for the dead, monuments to eternity: the deliberate destruction of megalithic graves by fire in the interior highlands of Iberia (Soria, province, Spain). *Oxford J. Archaeol.* **29**(3), 253-275 (2010). https://doi.org/10.1111/j.1468-0092.2010.00348.x

57. Bayliss, A. & Bronk-Ramsey, C. "Pragmatic Bayesians: a decade of integrating radiocarbon dates into chronological models" in *Tools for constructing chronologies: tools for crossing disciplinary boundaries*, C. Buck, A. Millard, Eds. (Springer, London. 2004), pp. 25-41.

58. Inizan, M.L., Roche, H. & Tixier, J. Avantages d'un traitement thermique pour la taille des roches siliceuses. *Quaternaria*. **19**,1-18 (1975).

59. Masson, A. Le comportement thermique du silex: application. *Staringia*. **6**(1),96-98 (1981).

60. D. R. Griffiths *et al*. "Experimental investigation of the heat treatment of flint" in *The Human Uses of Flint and Chert. Proceedings of the Fourth International Flint Symposium Held at Brighton Polytechni*, G. Sieveking, M. H. Newcomer, Eds. (Cambridge University Press, 1987), pp. 43-51.

Supplementary Table S1-S3

LAB NUMBER	CONTEXT	MATERIAL	BP DATE	CAL. BC 2σ (95.4%)	δ13C (‰)	δ15N (‰)	C:N	REFERENCE
Ua-65428	SU 39	Human bone (*Humerus*)	4973±33	3907-3650	-19,3	3,2	3,2	Unpublished
Ua-67323	SU 57	Human bone (*Tibia*)	4812±31	3645-3528	-19,8	10,2	3,2	Unpublished
Ua-65429	SU 27	Human bone (*Cranium*)	4550±32	3371-3102	-15,2	-	-	Unpublished
Ua-65425	SU 10	Human bone (*Cranium*)	4500±32	3356-3092	-19,5	8,5	3,3	Unpublished
Ua-61608	SU 4	Human bone (*Cranium*)	4490±35	3351-3033	-19,7	9,6	3,2	Unpublished
Ua-65426	SU 4	Human bone (*Femur*)	4484±32	3346-3031	-19,4	10,1	3,3	Unpublished
Ua-65427	SU 66	Human bone (*Cranium*)	4459±32	3339-3016	-20,0	3,4	3,2	Unpublished
Ua-61609	SU 18	Human bone (*Cranium*)	4448±42	3338-2930	-19,8	8,9	3,2	Unpublished
Ua-61610	SU 22	Animal bone (*Bos*)	4431±35	3331-2923	-21,1	6,8	3,3	Unpublished

Table S1. 14C dates from the Dolmen of El Pendón with lab references numbers (Ua=Tandem Laboratory, Uppsala University), the stratigraphical context of origin, the type of dated material dated, and the isotopic data –in case it is available-.

EL PENDÓN PASSAGE GRAVE (SEQUENTIAL MODEL)<br>Amodel = 95.6 /Aoverall = 77.1					
		**14C yr BP**	**MODELLED CAL. yr BCE**		**A (individual agreement indices)**
			**1σ (68.2%)**	**2σ (95.4%)**	
**PENDÓN III**	**End Pendón III**		3255-2877	3319-2708	
	**Span Pendón III (yr)**		0-5	0-5	
	Ua-61610	4431±35	3105-2936	3322-2922	106.7
	**Start Pendón III**		3265-3016	3356-2941	
	**Interval Pendón II-III (yr)**		0-122	0-253	
**PENDÓN II**	**End Pendón II**		3484-3102	3485-3071	
	**Span Pendón II (yr)**		0-84	0-178	
	Ua-61609	4448±42	3485-3164	3488-3102	87.4
	Ua-65427	4459±32	3485-3164	3487-3100	91.4
	Ua-65426	4484±32	3485-3163	3487-3111	94.5
	Ua-61608	4490±35	3486-3163	3487-3112	93.8
	Ua-65425	4500±32	3486-3164	3487-3114	90
	Ua-65429	4550±32	3486-3142	3488-3111	69.8
	**Start Pendón II**		3490-	3496-3143	

			3239		
	**Interval Pendón I-II (yr)**		59-294	0-382	
**PENDÓN I**	**End Pendón I**		3635-3473	3644-3324	
	**Span Pendón I (yr)**		18-149	9-221	
	Ua-67323	4812±31	3645-3536	3648-3531	96.7
	Ua-65428	4973±33	3742-3651	3785-3647	99.6
	**Start Pendón I**		3805-3661	4037-3648	

Table S2. Data table corresponding to the *Sequential phases* model. The results obtained through the *Span* and *Interval* commands have been included, which estimate the duration in years of each phase and the period between the estimated end boundary of one phase and the estimated start boundary of the next one, respectively.

EL PENDÓN PASSAGE GRAVE (CONTIGUOUS MODEL) Amodel = 105.2/Aoverall = 105.9					
		14C yr BP	MODELLED CAL. yr BCE		A (individual agreement indices)
			1σ (68.2%)	2σ (95.4%)	
PENDÓN III	**End Pendón III**		3256-2831	3308-2480	
	**Span Pendón III (yr)**		0-5	0-5	
	Ua-61610	4431±35	3262-2973	3290-2926	109.4
	**Transition Pendón II-III**		3292-3066	3322-3027	
PENDÓN II	**Span Pendón II (yr)**		0-133	0-229	
	Ua-61609	4448±42	3330-3158	3340-3085	100.5
	Ua-65427	4459±32	3329-3159	3339-3088	104.4
	Ua-65426	4484±32	3332-3158	3339-3101	106.5
	Ua-61608	4490±35	3334-3158	3341-3101	105.9
	Ua-65425	4500±32	3336-3154	3342-3104	102.3
	Ua-65429	4550±32	3359-3127	3364-3105	84.5
	**Transition Pendón I-II**		3391-3239	3496-3104	
PENDÓN I	**Span Pendón I (yr)**		78-203	17-249	
	Ua-67323	4812±31	3641-3533	3645-3528	98.8
	Ua-65428	4973±33	3763-	3797-3648	102.1

			3654		
	**Start Pendón I**		3920-3668	4469-3652	

Table S3. Data table corresponding to the *Contiguous phases* model. The results obtained through the *Span* command have been included, which estimates the duration of each phase in years.

Supplementary Figures S1-S11

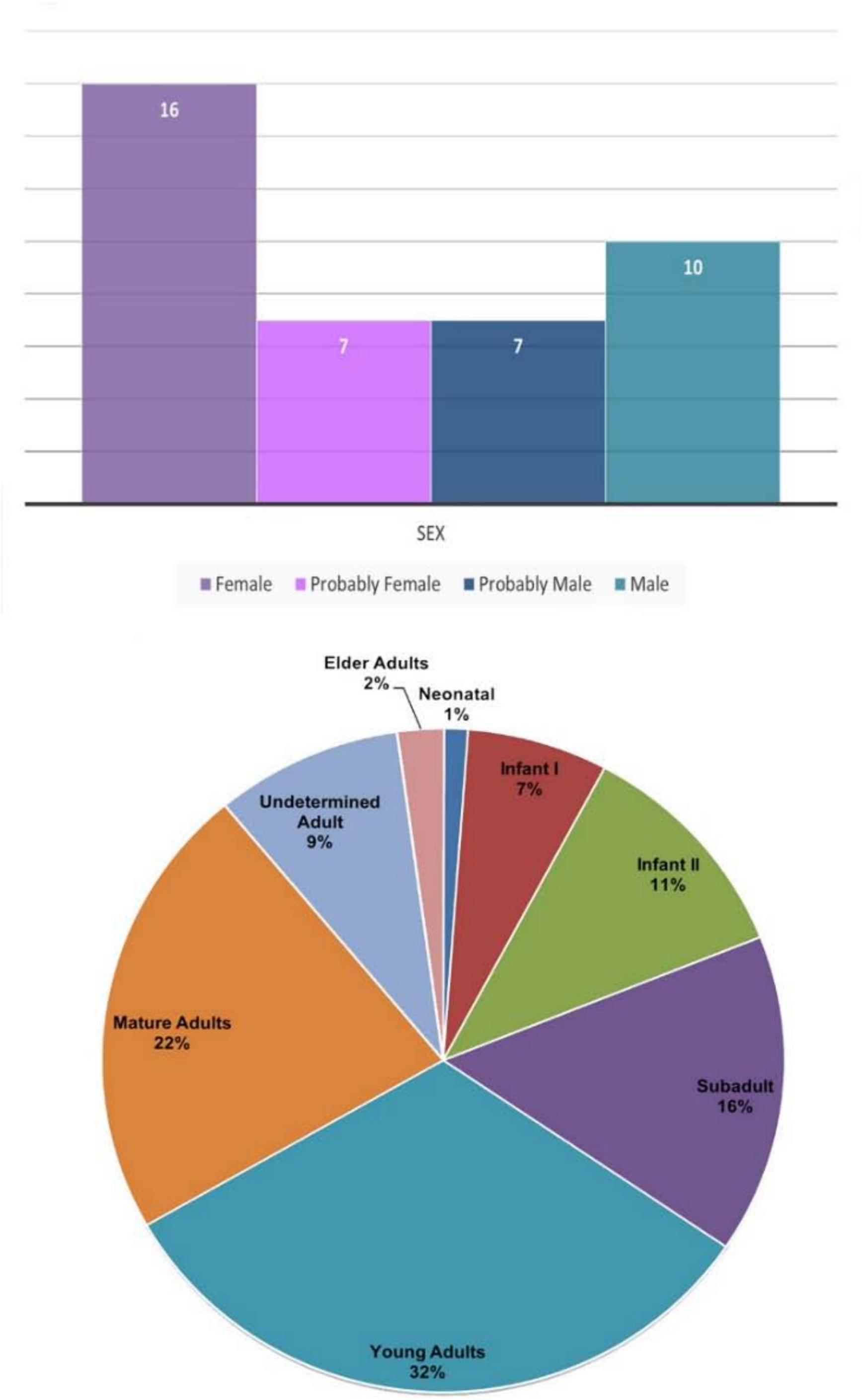

Fig. S1. Preliminary paleodemographic profile of the Dolmen of El Pendón. Superior: Graph of the MNI (minimum number of individuals) classified by sex. Inferior: Graph of the percentage distribution of individuals by age groups.

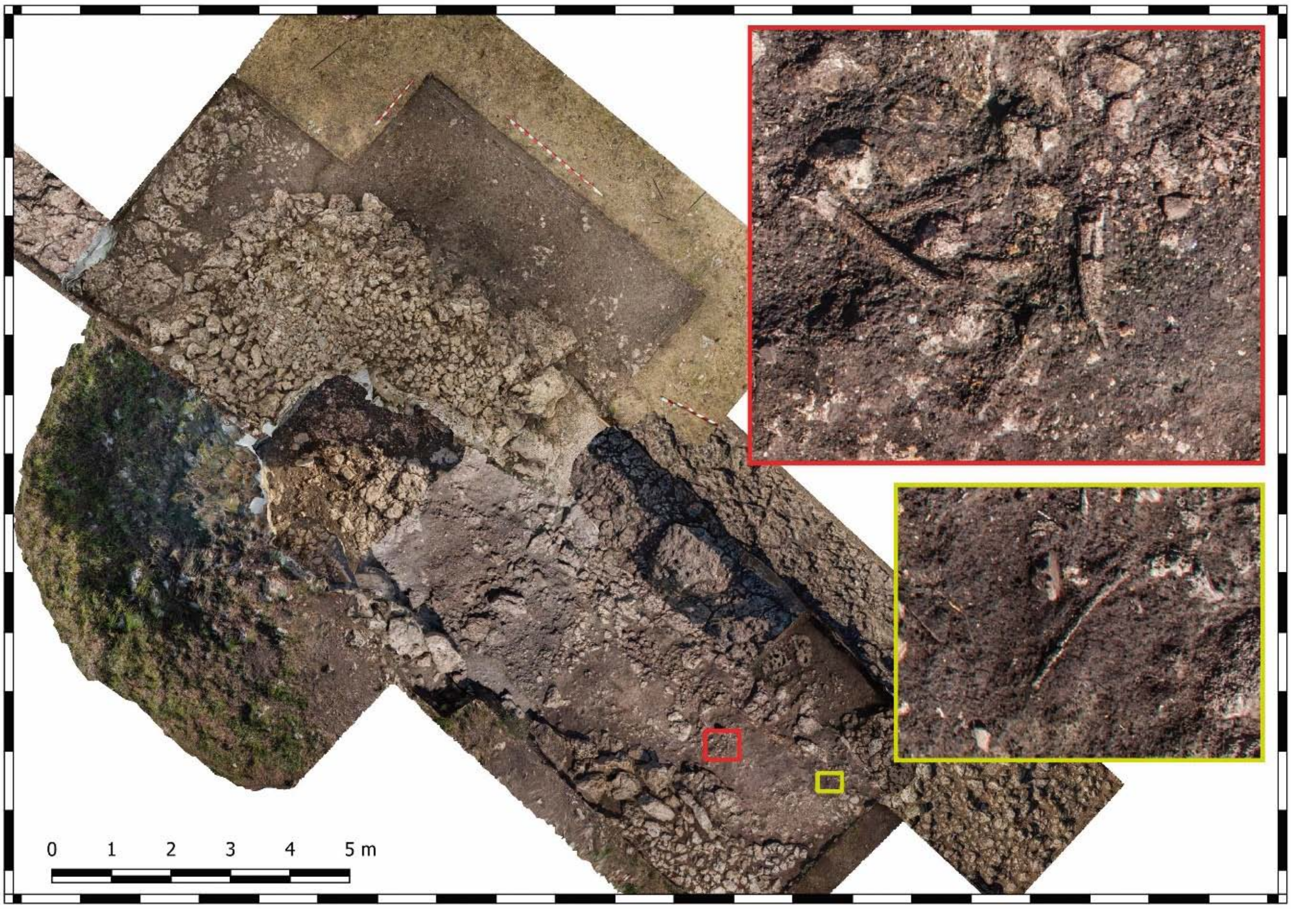

Fig. S2. Photogrammetric plan of the earliest levels of the Dolmen of El Pendón. In the enlarged images, the deteriorated look of human bones can be observed, which have provided the earliest dates of this megalithic monument.

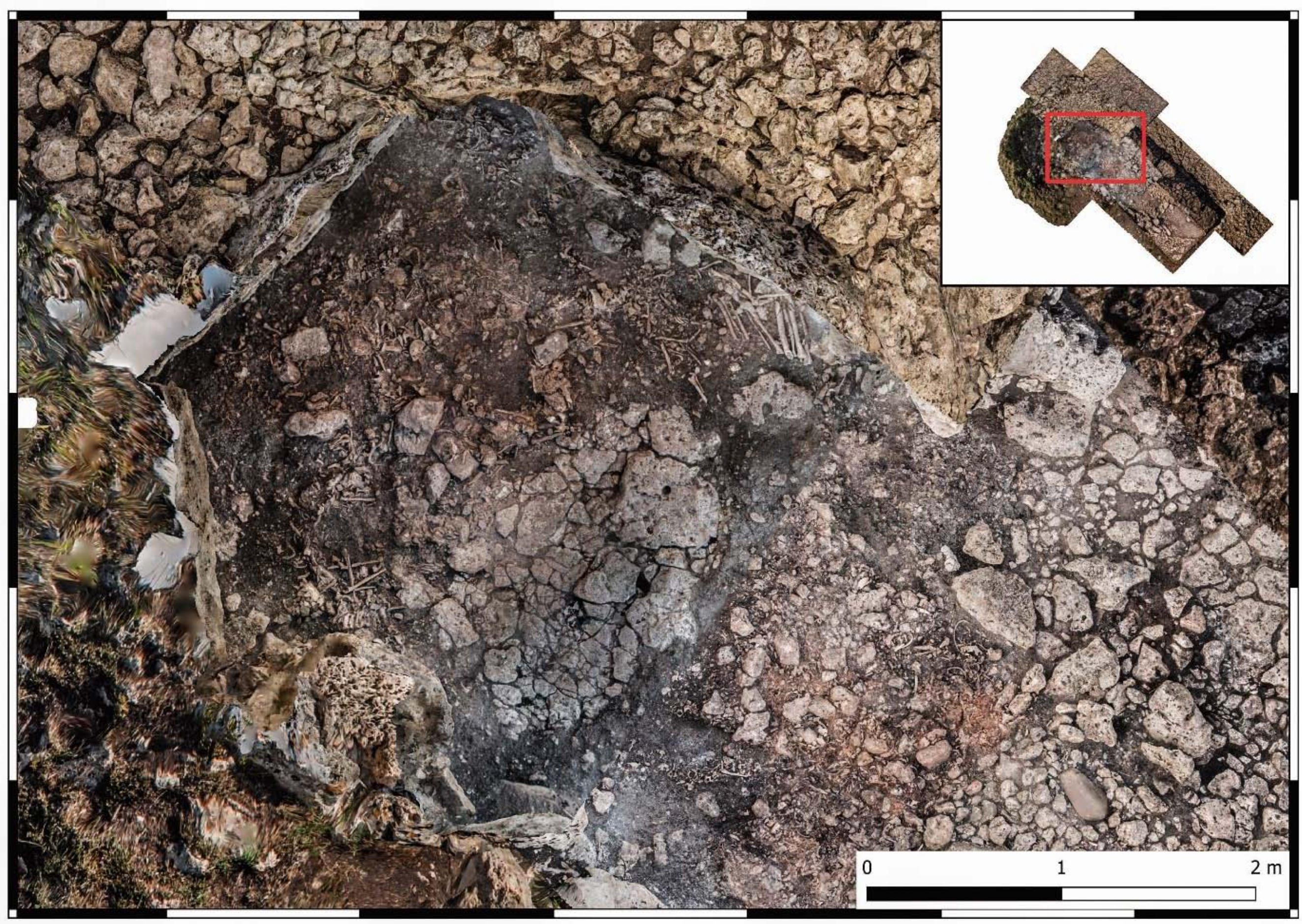

Fig. S3. Photogrammetric plan of the lower ossuary level (SU4) of the Dolmen of El Pendón.

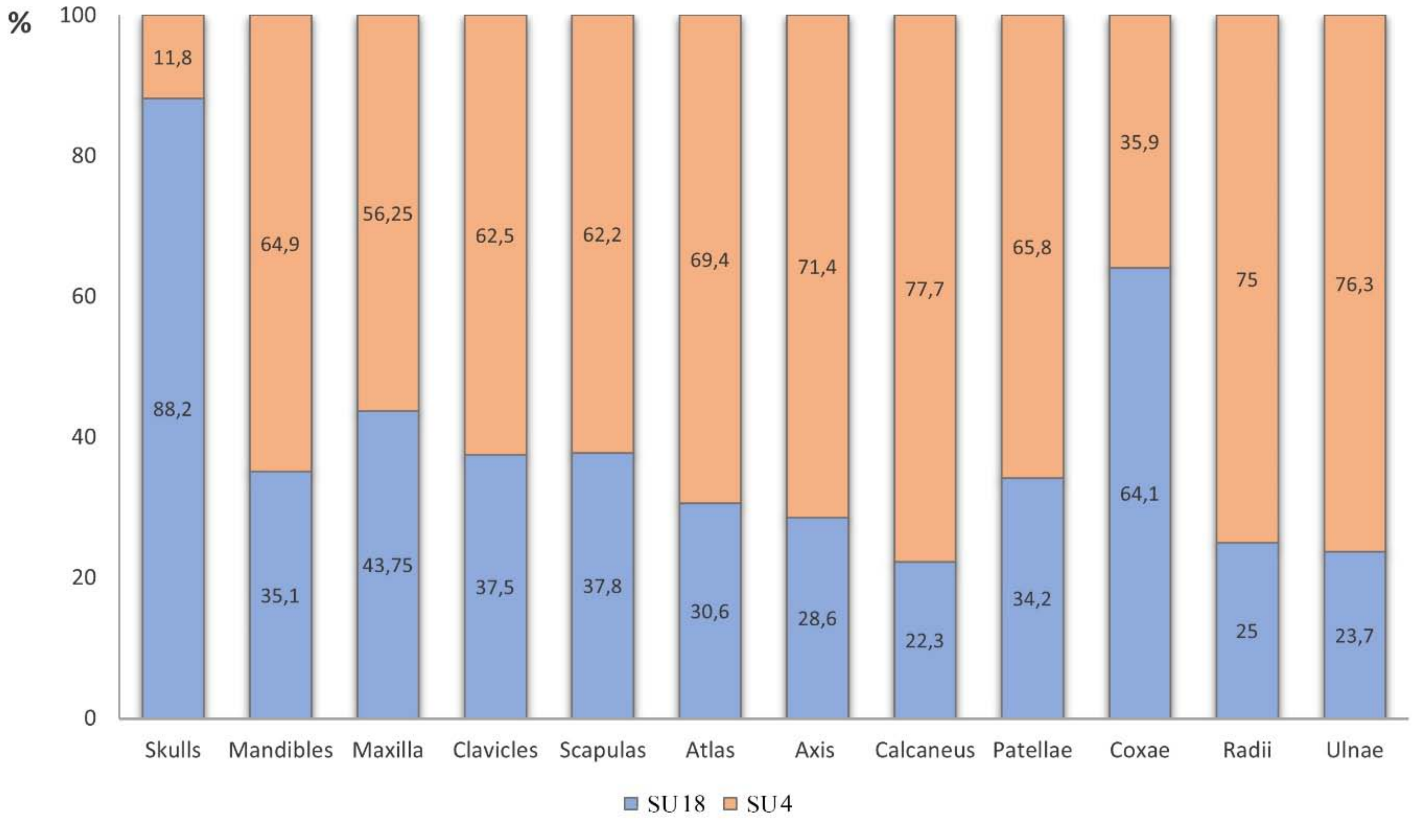

Fig. S4. Graph of skeletal representation in the two occupation levels of the burial chamber of the dolmen of El Pendón. Percentage distribution of the human bones remains by anatomic region documented in SU 4 (orange) and in SU 18 (blue).

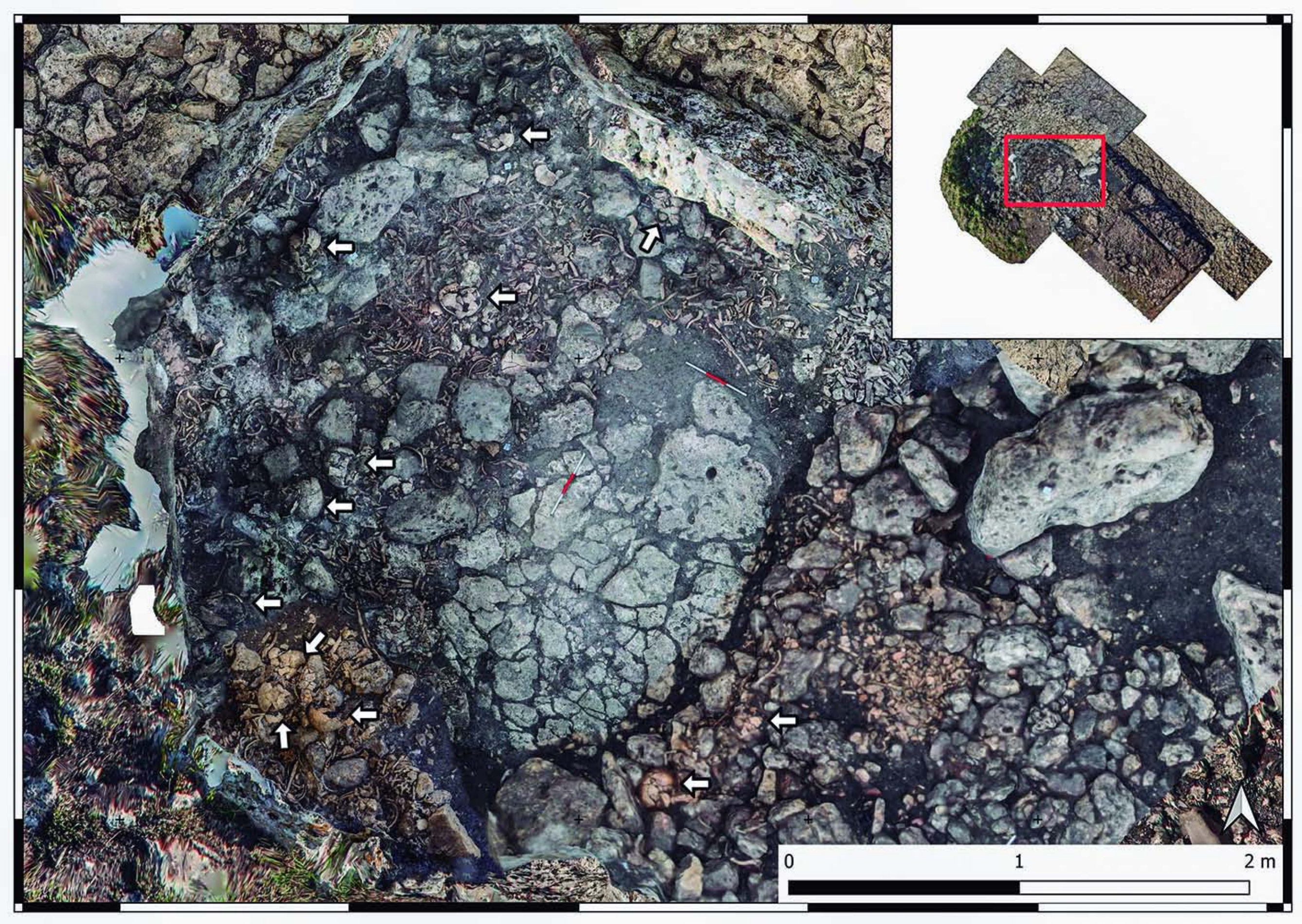

Fig. S5. Photogrammetric plan of the upper ossuary level (SU18) of the Dolmen of El Pendón. The arrows indicate some of the complete *in situ* skulls recovered during the excavation.

Fig. S6. Photogrammetric 3D model of the skull under study in this paper (by Santiago Sánchez de la Parra-Pérez). The 3D model is available in: https://sketchfab.com/3d-models/craneo-con-colesteatoma-del-dolmen-el-pendon-b4db066c84224a0f89701fe792563a77

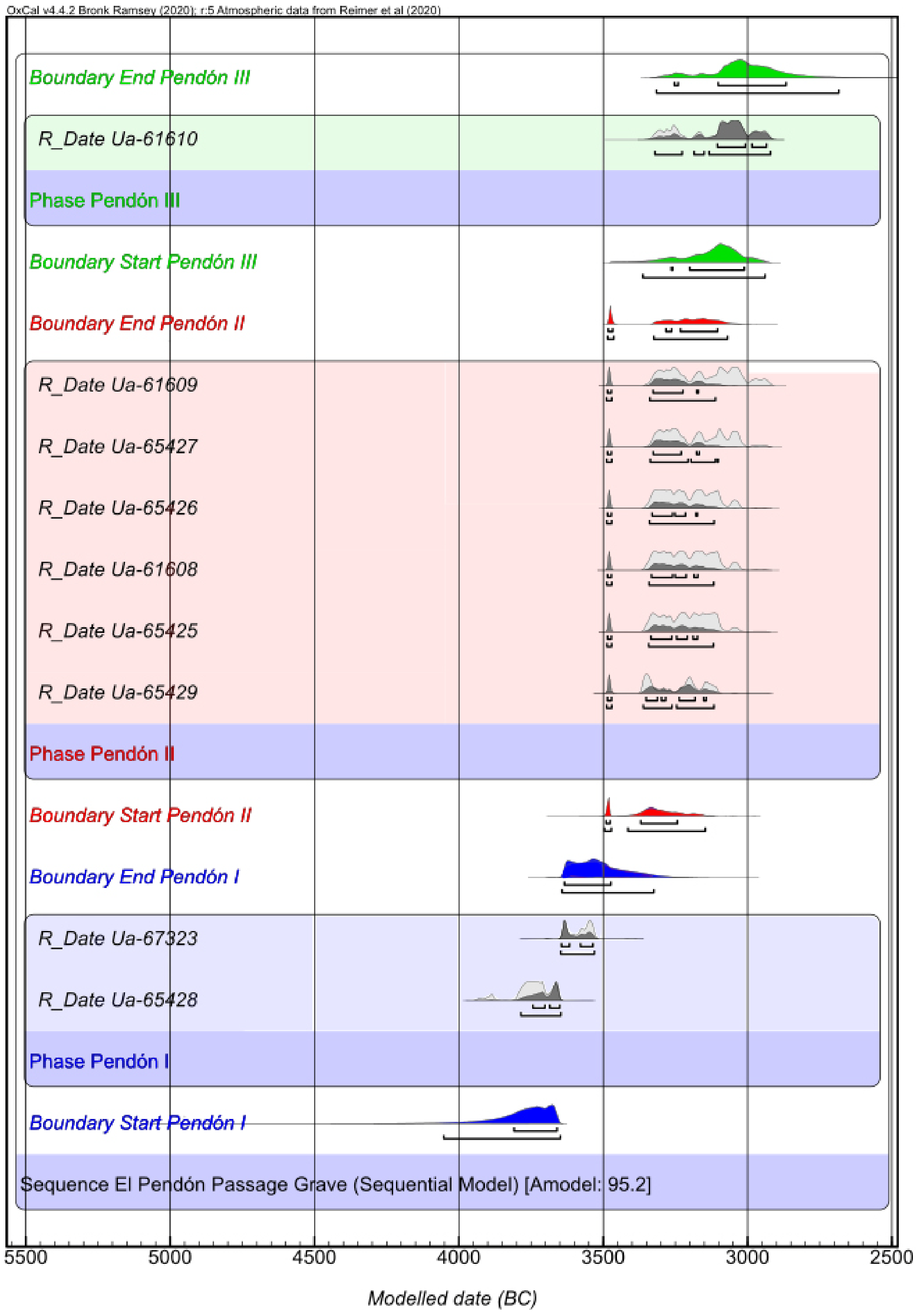

Fig. S7. Bayesian model *(Sequential phases analysis)* for the 14C dates from the Dolmen of El Pendón. Boundary estimates for each phase of megalithic activity are presented.

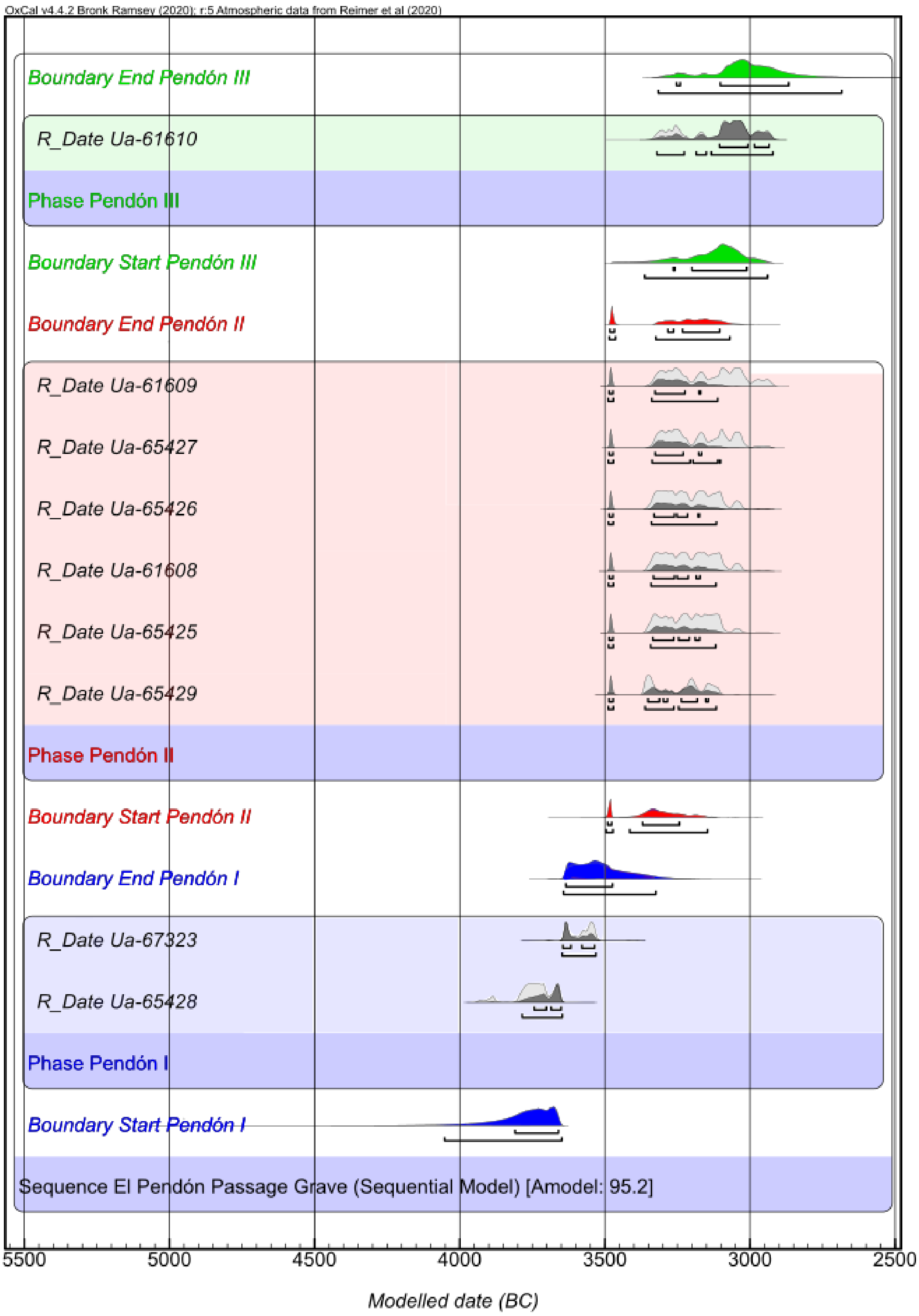

Fig. S8. Bayesian model (*Contiguous phases analysis*) for the 14C dates from the Dolmen of El Pendón. The estimated intervals for the transition periods between phases (black) are presented.

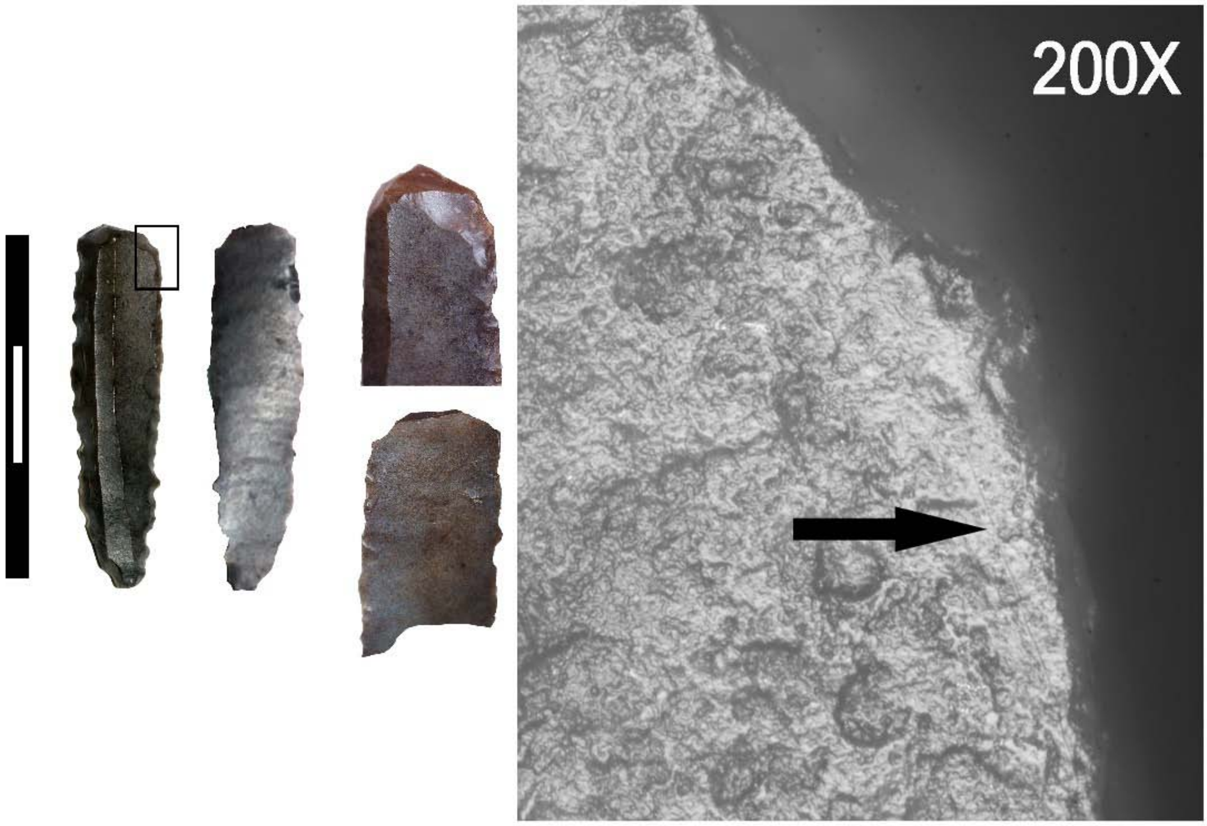

Fig. S9. Flint blade used in butchering tasks found in the ossuary of the Dolmen of El Pendón. On the distal part, there is an area with compact polish and striation marks with a diagonal orientation (visible at 200X).

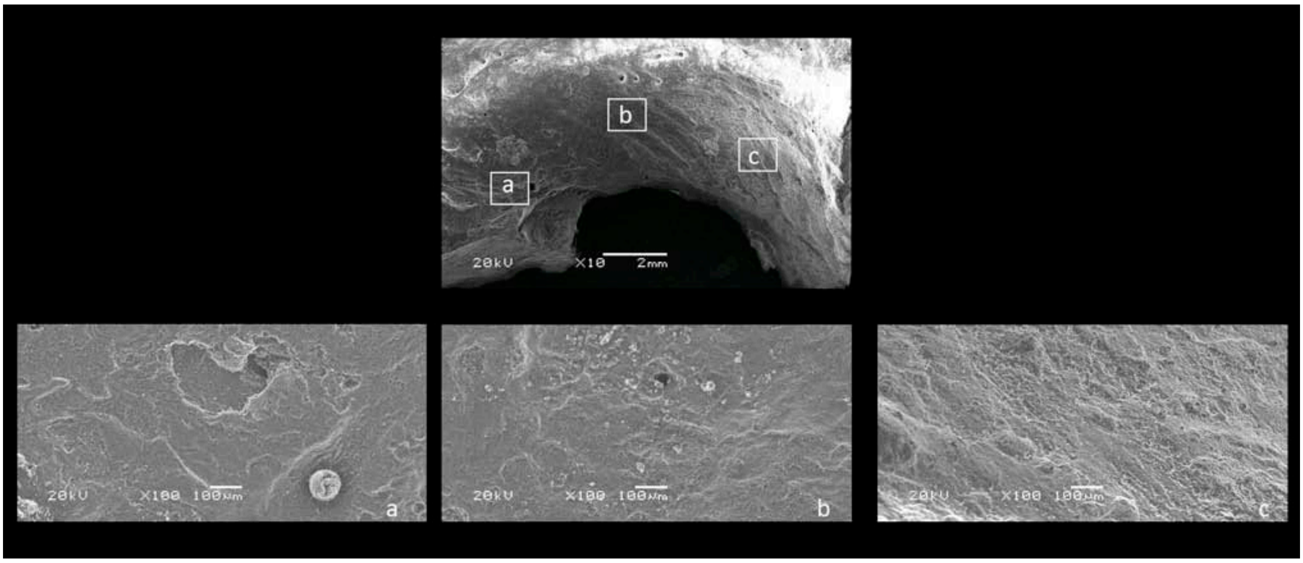

Fig. S10. Results of the surface histological analysis. Superior: Area of intervention in the left ear taken with a SEM (10X) -Anterior: left side of the image, Posterior: right side of the image-. Each square represents the zone where remodelling activity was recorded: A transition zone from a well-defined Howship's lacuna at upper center to a resting resorptive surface at bottom (a), a resorptive zone (b), and another transition zone from a resting resorptive surface at left to a resorptive zone at right (c).

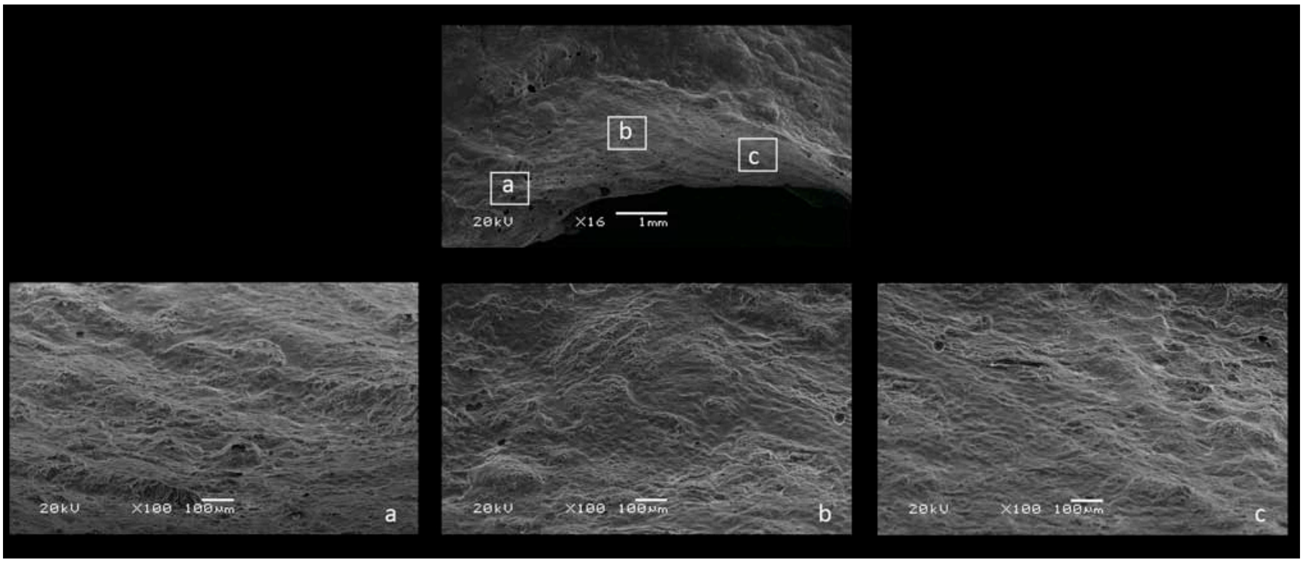

Fig. S11. Results of the surface histological analysis. Superior: Area of intervention in the right ear taken with a SEM (16X) -Anterior: left side of the image, Posterior: right side of the image-. Each square represents the zone where remodelling activity was recorded: A resorptive zone (a), a depository area (b), and the meeting edge of the deposition at left and resorption of bone at right along which the remodelling reversal is well defined.

Supplementary Video

Supplementary Video Legend: Explanatory presentation video of the Dolmen of El Pendón and the skull under study.
